# An Analysis of Regularized Second-Order Energy Expressions in the Context of Post-HF and KS-DFT Calculations: What Do We Gain and What Do We Lose?

**DOI:** 10.1021/acs.jctc.4c01547

**Published:** 2025-03-06

**Authors:** Igor Sawicki, Vincenzo Triglione, Subrata Jana, Szymon Śmiga

**Affiliations:** Institute of Physics, Faculty of Physics, Astronomy and Informatics, Nicolaus Copernicus University in Toruń, ul. Grudzia̧dzka 5, 87-100 Toruń, Poland

## Abstract

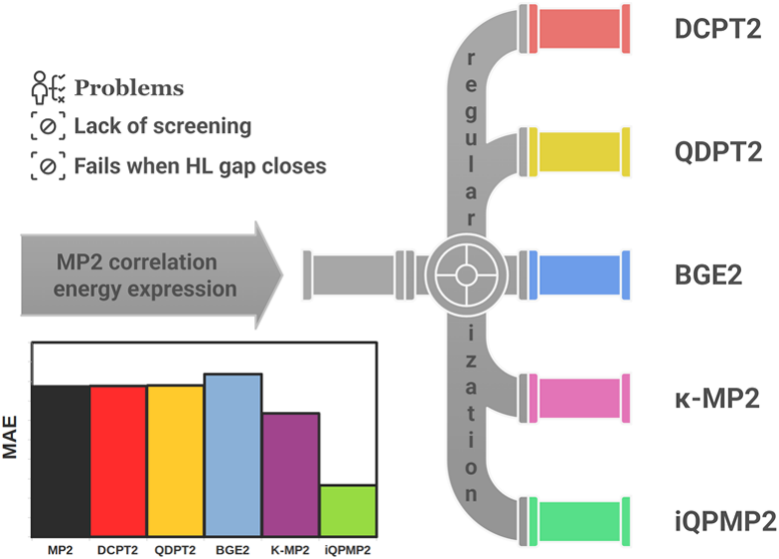

Møller–Plesset second-order (MP2) perturbation energy expression has been a workhorse for quantum chemistry methods for many years due to its very appealing accuracy/cost ratio compared to more advanced methods. It has been widely utilized in the post-Hartree–Fock (post-HF) calculations and Kohn–Sham density functional theory (KS-DFT) to define, e.g., the double-hybrid class of density functional approximations. Although the list of successful applications of the MP2 method is quite long, it suffers from various limitations, e.g., in strongly correlated systems, divergence in small energy gap systems, or overestimation of binding energies for large noncovalently bonded species. In this work, we analyze a few of the most commonly utilized forms of regularized MP2 correlation energy expression in the context of post-HF and KS-DFT calculations. To this end, we perform various tests for model systems, e.g., homogeneous electron gas, one-dimensional Hubbard model, Harmonium atoms, and some real-life examples, to trace back the advantages and disadvantages of these formulas, providing practical guidelines for their utilization in everyday quantum chemical calculations.

## Introduction

The second-order Møller–Plesset^1^ perturbation theory (MP2) energy expression is one of the first and most straightforward post-Hartree–Fock (HF) approaches to deal with electron correlation effects in many-electron systems. There exist many efficient implementations of the MP2 method^2^ in various quantum chemistry and solid-state packages, which allow to perform the correlated calculations with almost HF method scaling. This virtue and the fact that MP2 is size-extensive, size-consistent, and invariant concerning any unitary transformation among the occupied and/or virtual orbitals made it a very popular and attractive computational tool in the quantum chemistry realm. The second-order correlation energy expression has also been extensively utilized in Kohn–Sham density functional theory (KS-DFT), to define *ab initio* DFT,^3−8^ adiabatic connection models,^9−12^ or double-hybrid (DH)^13−15^ classes of exchange-correlation (XC) density functional approximations (DFA). In particular, the latter class of DFA has been proposed^13^ as an extension to standard hybrid functionals by incorporating the contribution from second-order correlation energy expression coming from Görling-Levy (GL2) perturbation theory^16,17^
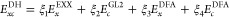
1where ξ_*i*_ are the parameters that scale all contributions, *E*_*x*_^DFA^ and *E*_*c*_^DFA^ denote here standard semilocal DFAs of exchange and correlation functionals, respectively, and *E*_*x*_^EXX^ is the HF (or exact) exchange energy
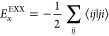
2The *E*_c_^GL2^ term is defined as a sum of single- and double-excited (*E*_D_^GL2^) contributions
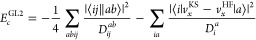
3where *D*_*i*···_^*a*_···_^ = ε_*a*_ + ··· – ε_*i*_ −··· are the denominators expressed in terms of KS orbital energies ε_*p*_, ⟨*ij*||*ab*⟩ being the antisymmetrized two-electron integrals in physical notation, whereas the indices *i*, *j* and *a*, *b* are used to denote occupied and virtual HF/KS orbitals, respectively. In the context of DH calculations, the single-excited term is usually neglected.^7,15,18^ The impact of this term is taken into account by the proper choice of the ξ_2_ parameter in eq 1. In the case of HF input quantities, eq 3 reduces to well-known MP2 correlation energy expression where the single-excited term vanishes due to Brillouin’s theorem.

Despite the context of utilization of eq 3, it possesses two noticeable drawbacks. The first emerges in the systems where the occupied and virtual orbital energy gap (highest occupied molecular orbital-lowest-unoccupied molecular orbital (HOMO–LUMO) gap) closes, causing the divergence of eq 3. This can be observed, e.g., in metallic systems^20^ or where strong correlation effects emerge.^21^ The second failure, in turn, is related to a substantial overestimation of the interaction energies in large polarizable systems. The origin of that is associated with the lack of inclusion of higher-order screening effects in eq 3,^22^ which causes significant overestimation (in some cases by more than 100%^22^) of interaction energies for large noncovalently bonded complexes. This feature causes the MP2 correlation energy expression to be unsuitable for the description of these types of problems.

Many useful strategies have been proposed to fix both deficiencies, e.g., rescaling of same-spin and/or opposite-spin correlation energies contribution in eq 3,^23−26^ utilization of correlated orbitals from orbital-optimized MP2^27,28^ or *ab initio* DFT methods,^4,7,8,29^ as well as attempts to include higher-order screening effects by utilization of adiabatic connection (AC) formalism.^30,31^

Another way to solve these drawbacks is the direct utilization in the post-SCF calculations of one of the regularized second-order energy expressions instead of eq 3. This allows, in some cases, partial incorporation of the higher-order screening effects at the second-order level and the avoidance of the singular correlation energies once the HOMO–LUMO gap tends to zero. To date, many valuable forms of regularized expression have been proposed^32−38^ and successfully applied in the context of post-HF calculations. Nonetheless, we remark that some of these expressions lead to new unwanted or even unexpected features, i.e., lack of size extensivity or size consistency, and vanishing correlation energy when the HOMO–LUMO gap tends to zero. A lack of detailed discussion about these deficiencies in the literature might hamper the future utilization of these expressions in various contexts. This is especially important for the KS-DFT framework, where error cancelation effects between semilocal and *ab initio* parts of second-order DFAs strongly impact the final performance of some approximations.^39^ Moreover, we recall that some of these expressions have already been utilized in constructing DH approximations.^40−42^ Thus, proper analysis can shed some new light on their current limitations and further improvements.

Here, we investigate five popular and recently utilized in post-SCF step^35,40−43^ regularized second-order energy expressions, showing their unknown similarities and main drawbacks that may impact routine, everyday quantum chemistry calculations.

The paper is organized as follows. First, we recall some main features investigated here, regularized second-order energy expression followed by their application in post-HF and KS-DFT calculations, respectively. More specifically, we perform various tests for some model systems, e.g., homogeneous electron gas (HEG), one-dimensional Hubbard model, Harmonium atom, and some real-life examples, to trace back the advantages and disadvantages of these formulas. We summarize the work with some practical guidelines for future applications in post-SCF calculations.

## Overview of Most Popular Regularized Second-Order Energy Expressions

In the literature, one can find many regularized second-order energy expressions such as the Epstein-Nesbet-type MP2 expressions,^44^ RD-MP2,^32^ REMP,^45^ AT-SCS-MP2,^25^ SRG(2)^46^ methods, and others.^30,33−38^ Because many of those formulas share some critical features, e.g., related to direct modification of the denominator or rescaling of eq 3 in our study, we have considered only the five most relevant expressions that have been recently applied both in the post-HF or KS-DFT calculations, and these are

(i) the second-order degeneracy corrected perturbation theory (DCPT) expression, which has been derived in ref (47) by considering the Hamiltonian of the two-level system at second order
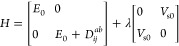
4where *V*_s0_ = ⟨*ij*||*ab*⟩, *E*_0_ is the unperturbed energy, and λ is the perturbation parameter. By performing direct diagonalization of eq 4 and summation over all doubly excited contributions, one obtains the DCPT2 energy formula
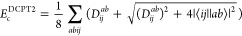
5Eq 5 is complete through second-order, containing a subset of higher-order terms. DCPT2 gives almost identical results to nondegenerate cases as the conventional MP2 energy expression. The improvement over the latter is visible in the cases of near-degeneracy situations, e.g., bond breaking.^47^ When the HOMO–LUMO gap goes to zero, the expression gives a finite contribution. Similarly to the MP2 method, the expression is size-consistent and invariant concerning orbital rotations. However, it is not size-extensive (will be discussed later).
(ii) Next expression is derived within quasi-degenerate perturbation theory (QDPT), taking as a starting point regular MP2 correlation energy expression with an additional shift of the denominator by the complex level shift parameter Γ_*ij*_^*ab*^^48^
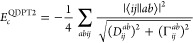
6Based on the choice of Γ_*ij*_^*ab*^, one can make the expression exact for two-level systems (Γ_*ij*_^*ab*^ = ⟨*ij*||*ab*⟩, like for the DCPT2 method) or by requiring that the Taylor expansion of the square root in eq 6 allows us to recover the fourth-order contribution (Γ_*ij*_^*ab*^ = 2 ⟨*ij*||*ab*⟩).^49^ The method is size-consistent^48^ but not size-extensive.^49^ Moreover, the method is not invariant w.r.t. orbital rotation.^48,49^
(iii) Another form of regularization is provided by the second-order Bethe-Goldstone (BGE2) energy expression^50^ where the denominator of eq 3 is supplied with pair-correlation energy (ε_*ij*_)
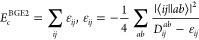
7which ensures that the correlation energy of any given system is finite. Despite this feature, eq 7 is size-consistent but not size-extensive and not invariant concerning orbital rotations, which might hinder its application.^40,50^ To calculate *E*_c_^BGE2^, one needs to solve eq 7 self-consistently, which is usually obtained within a few iterations. In the minimal basis set for H_2_ molecule^50^ BGE2 expression recover eq 5 (see eq 49 in ref (50)). We recall that in ref (40), the screened BGE2 (sBGE2) has been developed by introducing the semiempirical damping factor, which modifies the pair-correlation energy in eq 7 improving the description of dissociation of H_2_.^40^
(iv) The κ-MP2 regularized energy expression has been proposed in ref (35) by the introduction of a multiplicative dumping term dependent on empirical κ parameter and standard MP2 denominator. The κ-MP2 expression reads
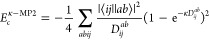
8and inherits most of the good features of the original MP2 method. Moreover, it does not diverge for systems where the HOMO–LUMO gap vanishes. However, in some cases, the dumping is too strong, leading to significant underestimation of correlation energy.^37,43^ We remark that similar expressions have been derived within the similarity renormalization group (SRG) approach described in ref (46) (see eq 24). In this study, we have utilized one of initial κ = 1.4 *E*_h_^–1^ parameter values, which was recommended for general application.^35^
(v) The last expression considered in this work was recently developed in ref (43) within quasi-particle (QP) MP2 theory (QPMP2). Here, we consider the iterative QPMP2 variant, denoted as iQPMP2, which takes the following form:
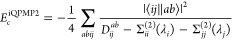
9with λ*_k_* being the QP orbital energies obtained by the self-consistent solution of Dyson equation in the diagonal approximation
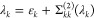
10with the Goldstone^43^ self-energy expression truncated at second order
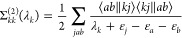
11The iterative process is usually done in a few cycles, taking as a starting point λ_*k*_ = ε_*k*_.^51^ Thus, the final occupied QP orbital energies are “dressed” in correlation effects, leaving virtual orbital energies intact. The expression is size-consistent and size-extensive. Moreover, although the original formulation utilizes the HF reference wave function, the orbital invariant formulation is possible.^38,43^ As was shown,^43^ this approach can be viewed as a size-consistent Brillouin-Wigner perturbation theory,^36^ which was proposed independently.^38^ We also underline that similar regularized MP2 energy expressions have been earlier studied in the context of the correlated one-particle method by Beste et al. in ref (52).

A summary of all of the most essential properties of the discussed expressions is given in Table 1.

**Table 1:** Summary of Discussed Regularized Second-Order Energy Expression Properties

	MP2	DCPT2	QDPT2	BGE2	κ-MP2	iQPMP2
size extensivity	yes	no	no	no	yes	yes
size consistency	yes	yes	yes	yes	yes	yes
invariance	yes	yes	no	no	yes	yes
iterative	no	no	no	yes	no	yes
thermodynamic limit (TDL)	no	no	no	no	partially	partially
strong correlation	no	partially	partially	partially	no	partially

## Computational Details

To assess the performance of the expression (i–v), we have conducted various types of calculations considering a few model systems and some real-life applications where MP2 usually exhibits problems. Because the primary purpose of the paper is the qualitative comparison and not the ultimate accuracy of regularized energy expression, most of the calculations have been performed in the TZ or QZ quality (depending on the size of the benchmark set) basis set without counterpoise (CP) corrections for the basis set superposition error (BSSE). The frozen core approximation was not applied in any case. If not specific elsewhere, all calculations have been performed in a locally modified version of PySCF^53^ program package, where all regularized energy expressions have been implemented. The computational details are listed below, and these are

- Homogeneous electron gas (HEG): These calculations are performed with a modified version of the UEGCCD program^54^ using the identical computational setup as in ref (37).
- Noncovalent interactions (NCI) data sets: The calculations for S22,^55^ S66,^56^ A24,^57^ and X31^38,58^ data sets have been performed using aug-cc-pVQZ^59,60^ basis set in the case of post-HF calculations, whereas for DH calculations performed in KS-DFT context, the def2-QZVP^61^ basis set was used.
- Harmonium atoms: We have performed calculations for various values of ω (∈ 0.03 ÷ 1000) in the Harmonium atom model^62^ using an identical computational setup as in our previous studies.^12,21,39^
- Hubbard model: We have performed calculations for one-dimensional, two and six sites, periodic half-filled Hubbard models^64^ with various interaction strength *U*/*t*.
- Dissociation curves: the dissociation of H_2_, C_2_, and N_2_ have been calculated using spin-restricted formalism. In the case of post-HF calculations, we have utilized aug-cc-pV5Z^65^ basis set for H_2_ and cc-pVTZ^66^ basis set for C_2_ and N_2_. In the case of DH KS-DFT calculations, def2-QZVP^61^ basis set was used.
- Termochemistry: as in ref (15), we consider selective test sets from the Minnesota 2.0 and GMTKN55^67^ data sets which represent overall essential chemical properties. The test sets are divided into three subsets, namely, main group thermochemistry (MGT), barrier heights (BH), and noncovalent interactions (NCI), and are listed in Table 1. In the case of post-HF calculations, we have used large aug-cc-pVQZ^59,60^ basis set. In the case of KS-DFT DH calculations, in turn, we have utilized the def2-QZVP^61^ basis set for all cases, except for the G21EA test set, for which the def2-QZVPD^61^ basis set is used.

## Results and Discussion

In the following, we assess and discuss the aforementioned regularized energy expression in the context of post-HF and KS-DFT calculations separately, underlining their advantages and their most severe disadvantages.

### Post-HF Calculations

#### Size Extensivity and Size Consistency

Any good quantum chemistry method should be size-extensive and size-consistent. Fulfilling these two fundamental properties is essential for molecular property calculations and applications to extended systems. We recall that for the size-extensive method, the total energy of the system *A* scales linearly with the number of atom *N*_at_ in the system *E*[*N*_at_*A*] = *N*_at_*E*[*A*]. For the size-consistent method, in turn, we require that the energy of two (*A* and *B*) infinitely separate species is equivalent to the energy of the whole system *E*[*A* + *B*] = *E*[*A*] + *E*[*B*].

**Figure 1:**
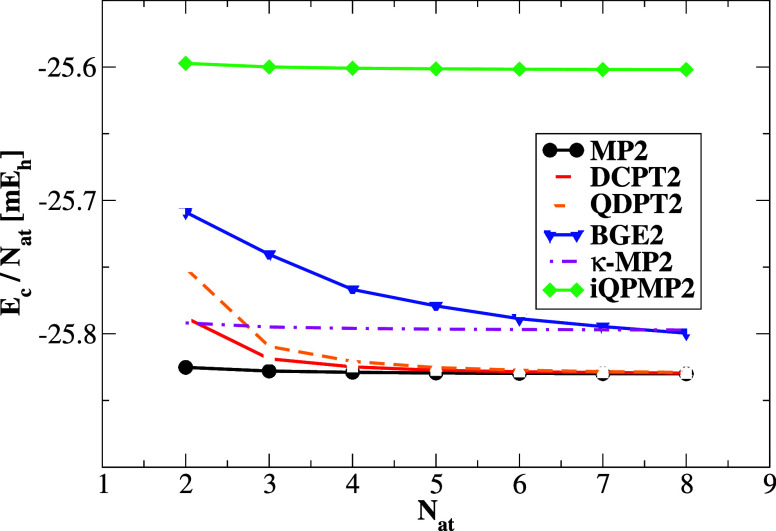
Correlation energy per atom in He chains of increasing size using the cc-pVDZ basis set as obtained for a few regularized energy expressions on top of HF reference orbitals. The spacing between He atoms was 3 Å. All calculations were corrected with a BSSE correction.

First, we focus on size extensivity; thus, in Figure 1, we report the correlation energy per atom in the chain of He atoms^37,38^ calculated in cc-pVDZ^66^ with BSSE correction. The calculations were performed on top of HF reference orbitals. The correlation energy per atom should have a zero slope for the size-extensive method, which is the case for the MP2, κ-MP2, and iQPMP2 methods. On the other hand, the BGE2, QDPT2, and DCPT2 regularizations are not size-extensive, which can be considered a notable drawback of these methods. This is especially important for BGE2 and DCPT2 expressions, which have been employed in the context of DH functional construction^40,41^ within the KS-DFT framework.

In the case of the DCPT2 and QDPT2 methods, the correlation energy per atom reaches the zero slope for a few He atoms. However, for finite systems, the size extensivity error is quite significant. In the case of the BGE2 method, the zero slope can be reached in the case of the infinitely large chain of He atoms that was already reported in the literature.^37,38^

All of the methods discussed here are size-consistent.^35,43,47,49,50^ However, several tests performed in this study revealed that DCPT2 and QDPT2 methods can give rise to nonphysical features in the interaction energy curves for weekly interacting systems. This issue is illustrated in Figure S1 of the Supporting Information (SI) (top panel), where we report the interaction energy curve for NeHe dimer computed using a cc-pVQZ^59^ basis set with BSSE correction. As one can note, around *R*/*R*_0_ ∈ (1.2, 1.8), the DCPT2 and QDPT2 methods produce a much larger potential barrier than other methods. The effect is related to small values under the square root  of both expressions. Utilization of larger basis sets, i.e., cc-pV5Z^59^ leads to the correct dissociation curve (see bottom panel of Figure S1 in the SI). Similar unwanted features have also been noted in many other basis sets, such as aug-cc-pVTZ^59,60^ or def2-QZVP.^61^ Thus, one should be careful about selecting the basis sets for these two methods. Another fact that could be noted is a slight underestimation of interaction energy for the iQPMP2 method, e.g., for the HeNe dimer, despite the basis set used. This is probably related to the larger QP HOMO–LUMO energy gap, leading to less attractive energy than the MP2 interaction.

#### Homogeneous Electron Gas Model

The homogeneous electron gas (HEG) belongs to the most fundamental models in solid-state physics, allowing the study of the properties of metallic systems.^68^ It is well known that MP2 correlation energy expression diverges for such systems when the number of electrons (*N*) tends to infinity (to the thermodynamic limit (TDL)).^20,69,71^ This is a consequence of the vanishing HOMO–LUMO gap in the MP2 denominator, leading to a large correlation contribution. We note, however, that utilization of higher-level methods in the same context leads to stable predictions^72,73^ for HEG.

The divergent behavior of MP2 energy expression^74^ also emerges in the calculations utilizing the finite simulation cell HEG model.^54,74,75^ As was shown in ref (74), the divergence persists even in the cases where the energy gap remains still wide open.^54,74,76^ This indicates the lack of effective screening of Coulomb interaction in MP2 expression, which is essential for the correct description of such systems.

**Figure 2:**
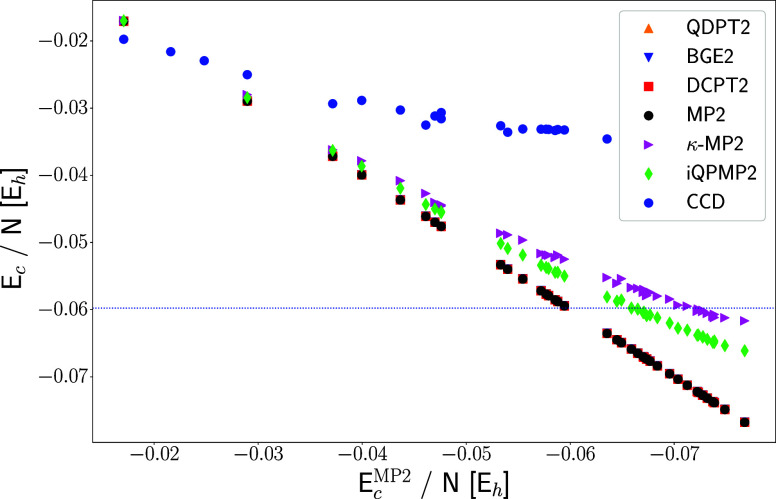
Correlation energy per electron for the HEG model (*r*_s_ = 1.0) computed for few regularized MP2 energy expressions in the function of MP2 correlation energy per electron. Deviation from linearity in this plot indicates convergence or slower divergence than MP2. The calculations have been performed using the HF reference state. The CCD data are taken from ref (76). For the basis set details, see ref (37). The horizontal line indicates the exact HEG correlation energy value computed using PW92^77^ correlation energy expression at the CBS limit.

Following ref (37), in Figure 2, we present the correlation energies for given regularized energy expression per electron in the function of the MP2 correlation energy per electron. All calculations have been performed using HF reference orbitals using identical computation setup as in ref (74). The deviation from linearity indicates convergence or slower divergence than the MP2 method.^37,76^ Each point in Figure 2 corresponds to a single HEG calculation for *N* ∈ (14, 3006) electrons in a finite cubic simulation cell with the Wigner-Seitz radius of *r*_s_ = 1 au. Figure 2 also includes the coupled cluster doubles (CCD) method results from ref (76) for comparison. In the SI, we additionally report similar data for *r*_s_ = 2.07 and *r*_s_ = 10.0 au. One can note that almost all expressions diverge, akin to the MP2 method crossing the exact HEG correlation energy per electron data (*E*_c_^LDA^/*N* ≈ −0.0598 *E*_h_) computed using Perdew and Wang energy expression (PW92).^77^ In particular, this is observed for QDPT2 and BGE2 methods, which regularize the gap and also DCPT2 energy expression, even though the closing HOMO–LUMO gap should not have a practical effect on this formula. This indicates that the origin of observed divergence (when *N* → ∞) is not related to the vanishing HOMO–LUMO gap but rather due to the lack of correct screening of integrals in the numerator. The latter point, in turn, can explain the behavior of κ-MP2 method for which the dumping factor (1 – e^–*κD*_*ij*_^*ab*^^)^2^ scales both the denominator and numerator of MP2 method making it less divergent with respect to exact HEG data. These findings align with observations made in ref (78). The iQPMP2 method, in turn, exhibits behavior similar to that of κ-MP2, being only slightly worse. This indicates that the utilization of QP orbital energies calculated by eq 10 can be considered as renormalization of the one-electron HF eigenvalues (by the inclusion of correlation effects) similar to the one performed for mosaic CCD method (see eq 16 in ref (76)). Because eq 10 also depends on the numerator of eq 3, this allows the gap to be opened significantly and stabilizes the numerator’s divergence. Thus, the results indicate that the QP orbital energies allow for partial inclusion of the higher-order screening effects in iQPMP2 energy expression.

A similar observation can be made for the case of more metallic densities (*r*_s_ = 2.07 and *r*_s_ = 10.0) reported in the SI. We see that the divergence persists for most MP2-like methods. This confirms the finding from ref (74). The κ-MP2 exhibits quite a large deviation from the linear divergent trend. For *r*_s_ = 10.0, in turn, κ-MP2 gives almost zero correlation energy. This is a consequence of closing the HOMO–LUMO gap, which causes the damping factor to vanish. On the other hand, the iQPMP2 energy expression exhibits very stable predictions despite the value of *r*_s_ showing its superiority upon the κ-MP2 method.

Thus, to conclude, we confirm the previous findings^37^ that fixing solely the problem with the HOMO–LUMO energy gap is insufficient to apply the regularized MP2 methods to describe metallic systems. The lack of description of higher-order screening effects by regularized energy expression hampers their utilization in this context. The summary of the performance of all methods in this context is shown in Table 1.

#### Large Benchmark Sets

As noted in ref (22), the lack of screening in MP2 correlation energy expression gives rise to significant relative errors, which can grow systematically with molecular size. This, in turn, makes the MP2 method practically unsuitable for the description of large noncovalent complexes. In this context, in Table 2, we report the MAE obtained for a few NCI data sets, namely, S22,^55^ S66,^56^ A24,^57^ and X31.^38,58^ We underline that these data sets span a wide range of molecular sizes and interaction types.

**Table 2:** Mean Absolute Errors (MAE, in kcal/mol) of Various Second-Order Regularized Expressions Computed Using HF Reference State for Few Noncovalent Interaction Data Sets^a^

	MP2	DCPT2	QDPT2	BGE2	κ-MP2	iQPMP2
S22	1.66	1.67	1.69	2.28	1.34	**1.05**
S66	1.20	1.21	1.22	1.63	0.96	**0.78**
A24	0.31	0.32	0.33	0.56	0.25	**0.18**
X31	1.13	1.14	1.15	1.37	0.94	**0.83**

a All calculations were performed in the aug-cc-pVQZ basis set. The smallest MAEs are highlighted in bold style.

One can note that both DCPT2 and QDPT2 expressions do not improve over the MP2 data for all benchmark sets. This is very similar behavior to that of the data presented in the previous section. The BGE2 method, in turn, exhibits a lower performance, showing an average 0.4 kcal/mol increase in MAEs with respect to MP2 data. This is probably related to the large size extensivity error observed for this expression, which affects the calculation of dimer interaction energies included in all sets (see Figure 1). In turn, the κ-MP2 method yields quite a nice improvement everywhere. As shown in refs (38,78), the performance of the κ-MP2 method for these systems can be further improved by properly tuning the κ parameter, which makes this method adaptive to new computational problems. The best performance is visible for parameterless iQPMP2 expression. For this case, one can note, on average, 0.4 kcal/mol improvement in MAEs compared to MP2 data. The trends are very similar to those of the HEG model above, confirming that κ-MP2 and iQPMP2 methods most probably benefit from partial inclusion of the higher-order screening effects.

#### Strongly Correlation Systems

In this section, we try to answer the question of whether the regularized MP2 methods can be successfully applied to systems in which a strong correlation regime emerges.

##### Harmonium Atom

**Figure 3:**
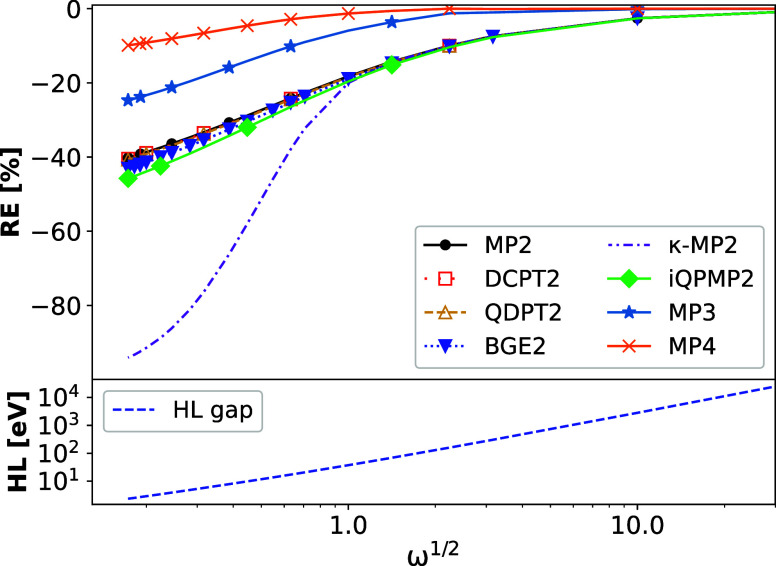
(Top) Relative error (RE = (*E*_c_^method^ – *E*_c_^FCI^)/*E*_c_^FCI^ × 100%) on correlation energies of harmonium atom in the function of ω calculated for the various second-order correlation energy expressions compared with MP3 and MP4 results. (Bottom) HOMO–LUMO gap energy. The calculations are performed on top of Hartree–Fock orbitals. The *x*-axis and *y*-axis of the HL gap panel are given on a logarithmic scale.

As a first example, we investigate the correlation energies for a two-electron Harmonium atom system, which is considered a simple but very fundamental test case for quantum chemistry methods.^12,21,79^ We recall that the system is strongly correlated for small values of ω, whereas for large values of the ω parameter, we enter the weekly interacting regime. This is reported in Figure 3, where we report the relative correlation energies computed with respected full configuration interaction (FCI) data on top of the HF reference.

One can note that all methods underestimate the results despite the value of ω. As shown, all expressions work similarly, being quite accurate in the weekly interacting regime (with 0.0% ≲ RE ≲ – 20%). In the strongly correlated regime (i.e., ω ≤ 0.5), akin to MP2, most second-order expressions underestimate the FCI correlation energy, yielding at ω = 0.03 RE between −40% ÷ −46%. The only exception from this trend is observed for the κ-MP2 variant, where for ω < 1, the error grows almost up to −100%. This effect results from a small dumping factor, which goes almost to zero with decreasing values of ω. The behavior of other MP2-like methods can be traced back to two reasons: (i) relatively large open HOMO–LUMO gap (see the bottom panel of Figure 3) even for the lowest value of ω (HL gap = 2.36 eV for ω = 0.03), which still does not produce divergence of MP2 expression; (ii) lack of inclusion of higher-order terms in the perturbation theory. One can note that the inclusion of the latter terms (see MP3 and MP4 data in Figure 3) results in much better prediction, especially visible for the strongly interacting regime. This suggests that the hereto-tested expressions do not catch this effect substantially to overcome the problems of the regular MP2 method.

We note that in general applications, the inclusion of higher-order MP*n* terms does not always lead to improved predictions.^22^ This is related to the slow convergence or divergence of the MP*n* series for some cases.^80^ Nonetheless, the higher-order terms can be partially included using adiabatic connection models to define correlation energy expressions,^12,21,30,81,82^ which are based on second-order terms.

##### Hubbard Model

A very good example of a model system that exhibits strong correlation features is the one-dimensional periodic Hubbard model,^64^ also studied recently in a similar context.^37,43^ Assuming the periodic boundary condition *c*_*N*+1σ_^†^ = *c*_1σ_^†^, the Hamiltonian can be written in the site basis as
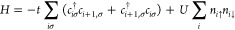
12where *c*_*i*σ_^†^ and *c*_*i*σ_ denote the creation and annihilation operators for electrons with spin σ at site *i*, respectively, whereas *n*_*i*σ_ = *c*_*i*σ_^†^*c*_*i*σ_ is the number operator. We recall that the behavior of this system is governed by the *U*/*t* ratio (where *t* is the hopping parameter and *U* is the on-site electron repulsion parameter), which provides a measure of the strength of the correlation. The system becomes strongly correlated for *U* ≫ *t*, providing an excellent tool for testing various electronic structure methods.

As in ref (43), we have utilized energy expressions to study the periodic half-filled Hubbard dimer and half-filled six-site Hubbard model in restricted HF configuration. For both models, the reference HF solution provides the finite constant HOMO–LUMO gap for any value of the *U*/*t* ratio.^43^ Hence, in this case, the divergence emerges due to a lack of effective screening of the Coulomb interaction in MP2 expression rather than the denominator.

**Figure 4:**
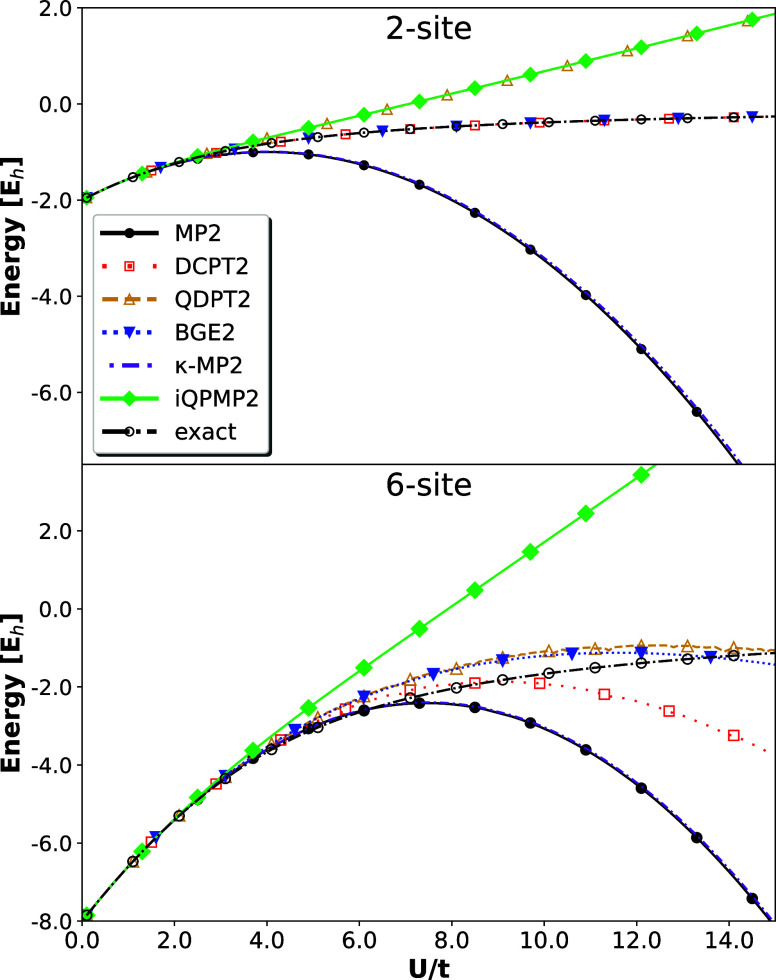
Ground state energy of the one-dimensional periodic six-site (top) and two-site (bottom) Hubbard model at half-filling for regularized expressions in the function of *U*/*t* ratio (*t* = 1.0). The calculations have been performed on top of HF reference orbitals.

In Figure 4 (top), we report the results obtained for a half-filled Hubbard dimer, which provides a simple prototype of H_2_ molecule in the minimal basis set.^83^ We report the data for several methods, including FCI. When *U*/*t* > 2, the MP2 starts to diverge due to the multireference character of the ground state wave function (well-known singlet–triplet instability^83^). The κ-MP2 method for standard parametrization (κ = 1.4 E_h_^−1^) follows the trend of MP2. This is because the dumping prefactor remains constant for all values of the *U*/*t* ratio. One can affect the behavior of the κ-MP2 method by changing the κ parameter value.^37,43^ However, this will shift only the divergence of energy expression to the larger values of the *U*/*t* ratio. What could be noted here is that BGE2 and DCPT2 methods recreate FCI data perfectly. This is not surprising since these two methods give identical energy expression^47,50^ in a minimal basis set equivalent to exact Hubbard dimer solution  We note that the exact solution is also provided by the QPMP2 method reported in ref (43), whereas the tween method, namely, the iQPMP2, overshoots the exact results significantly. QDPT2 energy expression, in turn, gives results identical to iQPMP2. This is because for occupied quasi-particle energy^84^ and unoccupied dimer orbital (ε_2_ = *U*/2 + *t*), the iQPMP2 expression reduces to QDPT2 one, meaning that in a minimal basis set, these two approaches are identical.

Similar behavior is also noted for the six-site periodic Hubbard model. κ-MP2 diverges as an unregularized MP2 method. Interestingly, the QDPT2 agrees quite well with FCI data. This is probably due to a *U*-dependent regularization factor in the denominator of QDPT2 energy expression, which significantly reduces the divergence. The second best agreement with reference data is provided by the BGE2 method, which also agrees with QDPT2 data up to *U*/*t* ≈ 11. For larger ratio values, these two energies start to differ. The BGE2 pair-correlation energy regularization factor is also *U*-dependent, meaning that the larger values of the *U*/*t* ratio give a dominant contribution in the denominator, leading to a reduction of overestimation. The DCPT2 energy expression also provides quite a nice divergence reduction up to *U*/*t* ≈ 10. The expression starts to diverge for larger ratio values, which can be easily seen from the form of eq 5. Last but not least, the iQPMP2 method, although it cuts off the divergence of the MP2 method, is quite inaccurate for a larger value of the *U*/*t* ratio compared to other methods.

##### Stretched Molecular Systems

**Figure 5:**
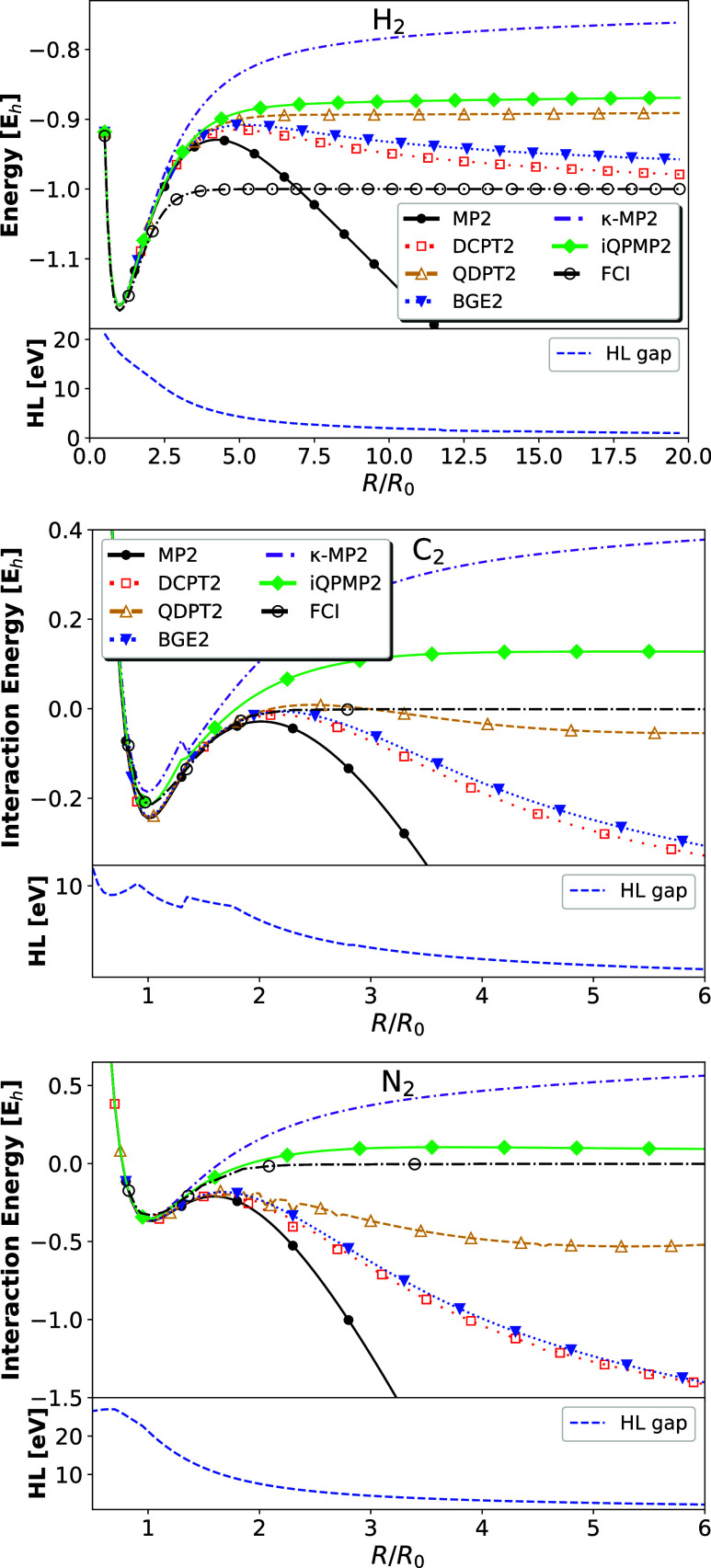
(Top) Total energy (for H_2_, top calculated in aug-cc-pV5Z) and interaction energies (for C_2_ and N_2_, middle and bottom panels calculated in cc-pVTZ), for three molecules as they are stretched, calculated with the various second-order correlation energy expressions obtained with HF orbitals using restricted formalism. The H_2_ FCI results have been obtained with aug-cc-pV5Z basis set, whereas C_2_ and N_2_ have been taken from ref (40). (Bottom) HOMO–LUMO (HL) gap energies.

Finally, let us focus on real molecular systems where strong correlation effects emerge. In Figure 5, we report the total energy (in the case of H_2_) and interaction energy (in the case of C_2_ and N_2_) as stretched along the interatomic distances using a restricted formalism.

First, we focused our attention on the H_2_ molecule. All methods give almost the same results for equilibrium distance, starting to differ for *R*/*R*_0_ > 2. We note that most methods stabilize the divergence of MP2 expression at a large *R*/*R*_0_ ratio. The worse performance is observed for the κ-MP2 method, which asymptotically goes almost to HF results.^43^ As previously mentioned, this is a consequence of closing the HOMO–LUMO gap (see bottom panel of the H_2_ figure), which causes the damping factor to vanish. We note that the proper behavior of the κ-MP2 method in this context can be restored by applying the machine learning techniques.^85^

Similarly to the half-filled Hubbard dimer, the QDPT2 and iQPMP2 methods perform very similarly, reaching at dissociation limits of ≈ −0.8865 *E*_h_ and ≈ −0.8616 *E*_h_, respectively. Interestingly, the curves of both methods are asymptotically parallel to the FCI reference. BGE2 and DCPT2 methods perform very similarly, agreeing up to *R*/*R*_0_ ≈ 4 with precursors and then going down asymptotically up to ≈ −0.9624 *E*_h_ and ≈ −1.015 *E*_h_, respectively.^86−88^ We recall that in a minimal basis set, these two methods yield identical results for H_2_.^50^ However, in this case, they go asymptotically to two different limits.

In the case of C_2_ and N_2_ molecules, which are excellent examples of breaking multiple bonds, the situation is very similar. At the equilibrium distance, all methods perform almost identically, except for κ-MP2, which underestimates the interaction energy for C_2_. One can note that around *R*/*R*_0_ = 1.6, κ-MP2 exhibits a slight kink in the potential of C_2_. A closer look at other methods reveals the same features but are more diminished. The kink is related to the wrong description of the interaction between the two lowest states, which was already reported.^40,89^ The same feature is also visible at the HOMO–LUMO gap presented in the bottom panel of C_2_. Generally, the best performance is observed for the iQPMP2 method, which gives at *R*/*R*_0_ = 6 the error of about 0.1 *E*_h_ for both cases. Notably, the method can break these two bonds smoothly using the RHF wave function, which is asymptotically identical to the UHF solution. As usual, the κ-MP2 gives a stable but unphysical behavior in the asymptotic limit. The BGE2 and DCPT2 methods, on the other hand, do not provide a qualitatively correct picture similar to MP2 expression. Lastly, the QDPT2 expression regularizes the MP2 divergence, leading to an underestimated but asymptotically stable prediction. We underline here that DCPT2, BGE2, and QDPT2 expressions are not size-extensive, which is most probably manifested in the interaction energy asymptotic behavior.

In conclusion, there is no universal second-order method that can work in all cases where signs of a strong correlation effect emerge. However, for real-life problems, the iQPMP2 method seems to provide the best reasonable choice to overcome the limitations of regular MP2 expression. The summary of the performance of all methods in this context is shown in Table 1.

#### Thermochemistry

In Table 3, we report the mean absolute errors (MAE) obtained for several thermochemistry benchmark data sets for investigated here regularized second-order energy expression. The results indicate (see total MAE–TMAE) that most of the expression (MP2, DCPT2, QDPT2) yield very similar predictions (TMAE ≈ 3.55 kcal/mol). However, few exceptions can be made. For example, in the case of the BGE2 method, we see a slight decrease in accuracy (TMAE *=* 3.68 kcal/mol), mostly due to problems with the accurate description of the NCI subset. This, in turn, could be related to the fact that BGE2 is fraught with the most significant size extensivity error. A different situation is observed in the case of κ-MP2 and iQPMP2 methods, which yield here TMAE of about 3.27 and 2.53 kcal/mol, respectively. One can note that the κ-MP2 method greatly improves the atomization energies (AE6, *G*/148 sets). This should not be surprising since this method’s κ parameter was optimized with respect to these energies. For other subsets, the method performs in line or worse than MP2. The most lousy performance is observed for the G21EA, which indicates the problems of the method in the description of the electron addition process. In the case of the iQPMP2 method, we see a large improvement in the case of MGT and NCI subsets, whereas, for the BH subset, we see an error increase of about 0.4 kcal/mol. Similar behavior was observed for the twin sBW2 method reported in ref (38) (see Figure 5). To conclude this part, most utilized regularized energy expressions yield inline or slightly better performance for the thermochemistry data set than the original MP2 method. The largest improvement is observed for the iQPMP2 method, which yields a TMAE of about 1 kcal/mol better than those of other counterparts.

**Table 3:** Mean Absolute Errors in kcal/mol for the Benchmark Tests Obtained Using HF Orbitals and Various Regularized Second-Order Energy Expressions^a^

	MP2	DCPT2	QDPT2	BGE2	κ-MP2	iQPMP2
main group thermochemistry (MGT)						
AE6	11.84	11.85	11.87	11.89	5.93	**3.93**
G2/148	11.86	11.86	11.88	12.01	7.33	**5.82**
G21EA	2.98	2.98	2.97	**2.94**	5.74	3.11
G21IP	4.26	4.25	4.23	4.21	4.68	**3.77**
PA26	2.25	2.25	2.26	2.26	1.75	**1.29**
BH76RC	2.63	2.63	2.63	2.66	2.56	**2.12**
SIE4 × 4	**1.56**	**1.56**	**1.56**	1.60	2.93	2.31

barrier heights (BHs)						
BH6	3.24	3.24	3.23	**3.18**	4.04	3.59
HTBH38	3.37	3.37	3.36	**3.26**	4.40	3.73
NHTBH38	5.20	5.20	5.19	**5.02**	6.32	5.48

noncovalent interactions (NCIs)						
HB6	0.35	0.36	0.37	0.89	0.56	**0.22**
DI6	0.97	0.98	0.99	1.17	**0.67**	0.69
CT7	0.96	0.99	1.02	1.20	**0.60**	**0.60**
PPS5	1.68	1.71	1.74	2.70	1.55	**1.28**
WI7	0.05	0.05	0.06	0.16	0.04	**0.02**
TMAE	3.55	3.55	3.56	3.68	3.27	**2.53**

a The last line reports the total mean absolute error (TMAE). All calculations have been performed in the aug-cc-pVQZ basis set. The best results for each set are boldface.

### KS-DFT Calculations

All DH DFAs inherit the drawbacks of standard second-order energy expressions (eq 1). To overcome these deficiencies, it is appealing to utilize the regularized second-order energy expression in the context of DH DFA construction.^40−42^ This is done in the current section for a few popular DHs, i.e., the first semiempirical B2-PLYP^13^ functional, the nonempirical PBE-QIDH^90^ and its range-separated variant RSX-QIDH,^91^ as well as BL1p^92^ DFA.

Since the iQPMP2 energy expression utilized the modified QP orbital energies, one must compute these energies for GKS DH reference orbitals. To this end, similarly as in refs (29,93,94), the second-order self-energy correction^95^ is calculated in post-SCF fashion on top of GKS orbitals (likewise for HF orbitals case^96^) and then the self-energy correction is rescaled by ξ_2_ parameter (see eq 1) and finally added to GKS counterparts to avoid double counting of correlation effects in orbital energies. We refer the reader to ref (93) for more details. In the case of the BL1p functional, the QP energies have been corrected as in the regular iQPMP2 method since this particular DFA utilizes the HF orbital set as an input.

#### Thermochemistry and Noncovalent Interactions

In Table 4, we present the TMAE values for the benchmark sets listed in Table 3, calculated using various DH approximations that incorporate the examined regularized MP2-like expressions.

**Table 4:** Total Mean Absolute Errors (TMAE) in kcal/mol for the Benchmark Set Obtained Using Various DH Functionals^a^

DH DFAs	%HF in (*E*_*xc*_, *v*_*xc*_)	MP2	DCPT2	QDPT2	BGE2	κ-MP2	iQPMP2
PBE-QIDH	(69%, 69%)	1.87	1.87	1.88	1.91	2.36	1.90
RSX-QIDH	(69%, 69%)	2.30	2.30	2.29	2.28	3.04	2.47
B2-PLYP	(53%, 53%)	1.99	1.99	1.99	1.99	2.52	2.07
BL1p	(82%, 100%)	1.81	1.81	1.81	1.86	2.85	2.28

a The second column reports the nonlocal HF contribution in *E*_*xc*_ and *v*_*xc*_, respectively. The full data for each DH are reported in the SI (Tables S1–S4).

One can note that for most DHs, there is practically no difference in the performance between the original MP2 and DCPT2, QDPT2, and BGE2 correlation energy expression, which yields almost the same TMAE for given DFA. These results should not be surprising for two reasons: (i) as for HF orbitals, also here for equilibrium geometries, the effect of regularization should be negligible; (ii) the second-order contributions in all DHs are always rescaled by ξ_2_ parameter (ξ_2_ < 1), which diminishes the impact of second-order term. A quite different situation is observed for κ-MP2 and iQPMP2. In the former case, we note an increase in the value of TMAEs, which is especially pronounced for RSX-QIDH and BL1p DFAs. This behavior should not be surprising since the κ parameter in the energy expression was tuned w.r.t. the atomization energies^35^ using HF orbitals. Thus, the κ value choice is not optimal for GKS orbitals of a given DFA. In the case of the iQPMP2 method, the largest decrease in accuracy can also be seen for RSX-QIDH and BL1p DFAs, which can be related to a modification of the GKS HOMO–LUMO gap by QP contributions, which causes an underestimation of the second-order term.

As noted in ref (42), to utilize the κ-MP2 energy expression in the context of DH functional, one needs to tune the value of the κ parameter. To test this possibility, for one representative DFA, namely, PBE-QIDH, we have optimized the κ parameter value with respect to the AE6 benchmark set leading to κ = 3.18 E_h_^−1^. The value is consistent with the one reported in ref (42). A similar procedure was applied for iQPMP2 energy expression, where the ξ_2_ (and thus ξ_4_ = 1 – ξ_2_) coefficient in eq 1 was amended. In this case, we obtained a ξ_2_ of 0.418, which is roughly 0.1 larger than the original parameters. Again, using those, we computed the TMAE for all benchmark sets from Table 3. This is reported in Table S5 in the SI. One can note that we recover a performance similar to that of unregularized MP2 energy expression for these tuned parameters. This confirms that the regularization does not drastically change the performance of DH DFAs for standard thermochemistry data sets. We note, however, that this behavior differs significantly from the one observed for HF orbitals (see Table 3), where the best iQPMP2 expression improved over the other formulas by ≈1 kcal/mol. This can be related to the fact that the performance of DH also depends on the mutual error cancelation effect between all terms in eq 1.^39^

As a further test of this part, we also employ the modified DH for noncovalent interaction data sets, as shown in Table 5. In this case, the situation is very similar to that observed in Table 4. In all cases, the DCPT2, QDPT2, and BGE2 variants perform almost in line with the original MP2-based DH. The κ-MP2-based method usually yields the same or poorer performance. The iQPMP2 DHs, in turn, seem to yield ambiguous behavior, giving an improvement for the A24 test set and slightly worse performance for the S22 and S66 sets. Utilization of reoptimized parameters (same as previously) in the case of PBE-QIDH DH for these last two expressions leads to results that are slightly better or in line with regular MP2-based DH (Table S6 in the SI).

**Table 5:** Mean Absolute Errors (MAE, in kcal/mol) for Few Noncovalent Interaction Data Sets Obtained Using Various DH Functionals^a^

	MP2	DCPT2	QDPT2	BGE2	κ-MP2	iQPMP2
PBE-QIDH						
S22	0.74	0.73	0.72	0.68	1.06	0.85
S66	0.81	0.81	0.80	0.74	1.05	0.88
A24	0.26	0.25	0.24	0.22	0.33	0.28
X31	0.61	0.60	0.60	0.58	0.76	0.65

RSX-QIDH						
S22	0.92	0.92	0.90	0.81	1.15	1.01
S66	0.96	0.96	0.94	0.87	1.13	1.01
A24	0.34	0.26	0.33	0.28	0.39	0.28
X31	0.72	0.72	0.71	0.69	0.83	0.74

B2-PLYP						
S22	1.79	1.76	1.74	1.49	2.02	1.87
S66	1.59	1.57	1.55	1.39	1.78	1.65
A24	0.50	0.48	0.46	0.39	0.56	0.27
X31	0.91	0.90	0.89	0.84	1.08	0.95

BL1p						
S22	0.24	0.25	0.25	0.34	0.48	0.42
S66	0.34	0.33	0.33	0.33	0.59	0.51
A24	0.24	0.18	0.17	0.21	0.27	0.24
X31	0.35	0.30	0.30	0.30	0.44	0.35

a All calculations were performed in the def2-QZVP basis set without BSSE correction.

**Figure 6:**
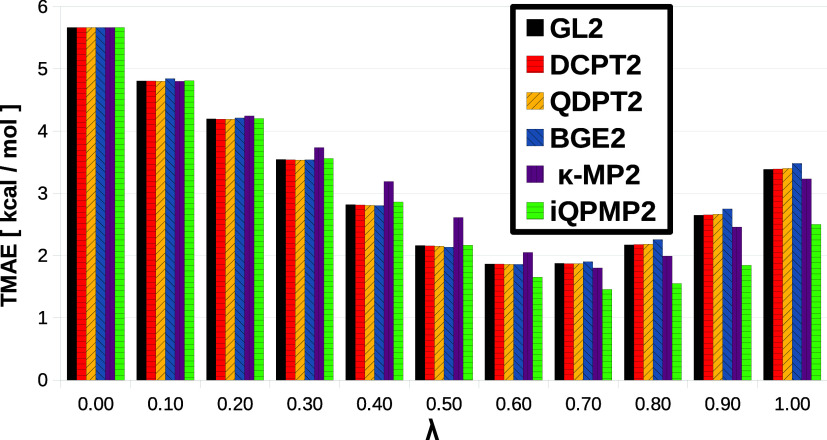
Total mean absolute errors (TMAE) (in kcal/mol) for the thermochemistry benchmark sets obtained for one parameter 1DH-BLYP DFA in the function of λ ∈ ⟨0, 1⟩ parameter (HF exchange contribution).

As a final example in this section, we test how the percentage of HF contribution in DH DFAs impacts the behavior of various regularized energy expressions for all benchmark sets from Table 3. To this end, we have utilized the one parameter BLYP-based DH functional (1DH-BLYP, which is very similar to tested here B2-PLYP and BL1p variants) expression^97^
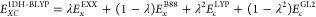
13computing the TMAEs in the function of λ ∈ ⟨0, 1⟩ parameter (HF exchange contribution). We remark that for the λ = 0 case, we recover the semilocal BLYP total energy expression. In contrast, for λ = 1, we get the total energy obtained for all expressions computed at HF orbitals. This is reported in Figure 6.

One can note that for λ < 0.2, all energy expressions work very similarly, giving almost identical TMAEs for a given λ. This is unsurprising since the ξ_2_ = λ^2^ parameter for this case is <0.04, which indicates the marginal impact of the second-order term and dominant role of semilocal correlation part of DFA.^93^ For the range of λ ∈ ⟨0.2, 0.6⟩, one can observe that κ-MP2 (with original κ = 1.4 E_h_^−1^ value) energy expression gives the worst predictions w.r.t. others for the same reasons as discussed above. This would also explain the quite bad performance of this energy expression for B2-PLYP DFAs (where the HF contribution is 53%, see Table 4). Nonetheless, the properly tuned κ parameter value should allow improvement of its performance in line with other expressions. In the same range of λ, the DCPT2, QDPT2, and BGE2-based DH perform almost identically to the original variants despite their problems with size extensivity. This is probably due to the mutual error cancellation effect between the semilocal and *ab initio* parts of DH energy expression. This could also explain the relatively good performance of BGE2-based DH functional defined in ref (40), which optimally tuned parameter λ = 0.5.

One can note that TMAE for all expressions reaches for λ ∈ (0.6, 0.8) its minimum, which is especially visible for the iQPMP2-based method. Because all energy expressions use the same 1DH-BLYP GKS hybrid orbitals and eigenvalues, the improved performance of iQPMP2 comes directly from the quality of utilized QP orbital energies. One can note that for λ = 0.8, the iQPMP2-based DH yield TMAE = 1.55 kcal/mol. At the same time, the BL1p containing a very similar 82% HF contribution in the *E*_*xc*_ formula gives TMAE = 2.28 kcal/mol. The difference in the behavior can be attributed to the different choice of reference orbitals, which, in the case of the latter method, is set to HF ones. Similar behavior can also be seen in the case of the κ-MP2-based expression. Also, here, we note the difference in the performance between the GKS and HF choice of the orbitals. This can indicate that the GKS orbitals can provide much more balanced error compensation between the orbital-, eigenvalue- and functional-driven errors^39^ in the 1DH-BLYP energy expression than HF counterparts.

Beyond the λ > 0.7, the TMAEs for all methods start to grow, reaching at λ = 1 similar performance as in Table 3 (100% of HF). In the same range, the BGE2 expression gave one of the worst TMAEs. The DCPT2 and QDPT2 expressions work almost identically with the initial MP2 variant. The most significant improvement in this range is observed for iQPMP2 energy expression, which yields TMAEs usually 0.3 ÷ 0.9 kcal/mol smaller than those obtained from the original MP2-based DH definition.

In conclusion, for this part, in the case of DH DFAs, for which the HF contribution is smaller than 0.6 (in both XC functional and potential), the recommended regularized energy expressions are DCPT2, QDPT2, and BGE2. The κ-MP2 expression can be utilized in a medium range of HF contributions with additional optimization of κ parameters. For larger HF contributions (in both *E*_*xc*_ and *v*_*xc*_), the most significant advantage is obtained for iQPMP2 energy expression, which can be recommended for these cases.

#### Molecular Bond Dissociation

**Figure 7:**
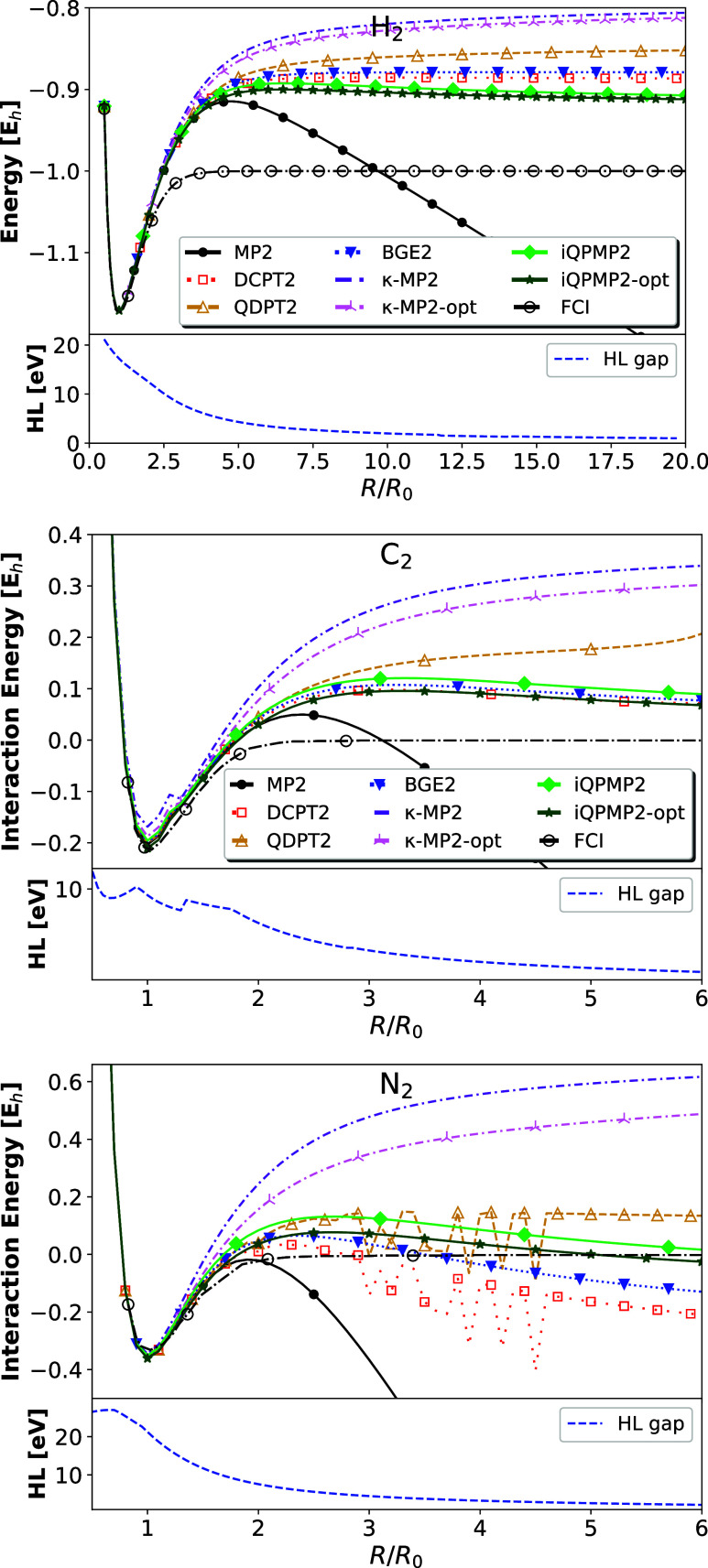
(Top) Total energy (for H_2_, top) and interaction energies (for C_2_ and N_2_, middle and bottom panels, respectively) for three molecules as it is stretched calculated with the various second-order correlation energy expressions obtained with the RHF reference. All calculations have been performed in the def2-QZVP basis set with PBE-QIDH functional. (Bottom) HOMO–LUMO (HL) gap energies.

In Figure 7, we applied the regularized PBE-QIDH-based energy expressions for bond-breaking problems in H_2_, C_2_, and N_2_ molecules with a restricted formalism. In the bottom panels, we report the HOMO–LUMO gaps obtained from GKS PBE-QIDH calculations.

First, we concentrate on the H_2_ molecule. One can note a similar behavior to HF orbitals; e.g., near the equilibrium distance, all expressions yield very similar results starting to differ for *R*/*R*_0_ > 2.5. The BGE2 and DCPT2-based DH give almost the same performance, very similar ZRPS DH functional developed in ref (40). The iQPMP2 base PBE-QIDH DH provides results that are in line with those of precursors, yielding slightly lower energy at dissociation. The κ-MP2, in turn, again gives quite different behavior due to the large dumping factor. Utilization of the reoptimized κ value only slightly improves its performance.

The same overestimation of κ-MP2-based DH is visible for interaction energies of C_2_ and N_2_ molecules reported in the middle and bottom panels of the same figure, respectively. In turn, the BGE2, iQPMP2-based expressions provide quite balanced descriptions at all bond distances very similar to the one reported in ref (40). On the other hand, the DCPT2 and QDPT2 DHs yield very unstable results, which are especially visible in N_2_ case. We have checked this effect, and we confirm that it emerges due to numerical instabilities related to the very small *D*_*ij*_^*ab*^ and ⟨*ij*||*ab*⟩ values under the square root in eqs 5 and 6. This effect was also visible in many other basis sets and DH energy expressions (not reported). Hence, we recommend the utilization of these two energy formulas with high caution.

## Conclusions

In the study, we analyze a few forms of MP2 correlation energy regularizations, namely, DCPT2, QDPT2, BGE2, κ-MP2, and iQPMP2 variants in the context of post-HF and KS-DFT calculations. To this end, we have performed various tests for model systems, e.g., HEG gas, one-dimensional Hubbard model, Harmonium atom, and some real-life examples to trace back the advantages and disadvantages of these formulas.

In the post-HF context, the DCPT2, QDPT2, and BGE2 energy expressions yield very similar performance to the regular MP2 counterpart despite their problem with size extensivity. This can be seen, e.g., for HEG gas, large noncovalent or thermochemistry data sets. These methods, in turn, give reasonably accurate results for the two- and six-site Hubbard models, removing the divergence of MP2. The best results in the post-HF calculations are yielded by the iQPMP2 method, which provides a significant improvement over MP2, also outperforming the κ-MP2 method in many cases. This is especially noticeable in the thermochemistry data sets and large benchmark sets, where, for the latter, the iQPMP2 method breaks the barrier of chemical accuracy. Moreover, it also yields very good results for systems with strong correlation (Hubbard model) and bond-breaking problems with static correlation, where usually the HOMO–LUMO gap closes (H_2_, C_2_, and N_2_). We remark that in the same situation, the κ-MP2 method provides unsatisfactory performance, leading to underestimation of energy at dissociation.

Concerning the application of the same formulas in the KS-DFT context, one does not observe any beneficial effect coming from their utilization for standard parametrization of any DH formula. In other words, the simple substitution of the MP2 correlation term with any regularized counterpart does not provide any improvement in their performance. Moreover, the utilization of κ-MP2 and iQPMP2 variants usually leads to a worsening of the results. However, one can see the improvement by simple tuning of the DH parameters, e.g., the κ and ξ_2_ in the case of κ-MP2 and iQPMP2, respectively, which allow to recover the initial performance.

Furthermore, we found that the quality of thermochemistry results strongly correlates with the portion of the nonlocal HF contribution in the given DH DFA. For functionals where the ξ_1_ parameter is smaller than 0.6 (both in XC functional and GKS potential), the best performance is obtained for DCPT2, QDPT2, and BGE2 energy expression despite their problems with size extensivity. The κ-MP2 expression can be utilized in a medium range of HF contributions with additional optimization of κ parameters. For larger HF contributions, the most significant advantage is obtained with the iQPMP2 energy expression, which can be recommended for that case.

To some extent, the utilization of regularized second-order expression leads to some improvements in the description of DH in the bond-breaking problems (see the examples of H_2_, C_2_, and N_2_ molecules), especially visible for BGE2 and iQPMP2 DH-based expression. The lack of size extensivity does not substantially impact the behavior of the former, and it is most probably compensated by a small scaling factor in the PBE-QIDH functional. The κ-MP2-based one usually underestimates the results, whereas DCPT2 and QDPT2 give some signs of numerical problems related to the square root present in their formulas. Thus, these two latter expressions are not recommended in any application of this kind.

In conclusion, all data show that there is no universal regularized second-order method that can work in all investigated here cases. However, for real-life problems, the iQPMP2 method seems to provide the best, reasonable choice to overcome the limitations of regular MP2 expression in both post-HF and KS-DFT contexts. Our final recommendation is that one should definitely avoid expressions that lack basic properties, e.g., size extensivity, etc., which may hamper the performance of new methods or lead to the wrong interpretation of final data.

As a future direction, we plan to compare the performance of regularized MP2 methods to extended systems. This could be mostly interesting in the context of metallic systems, where regular MP2 expression utilization is very challenging.

## Data Availability

The data that support the findings are published within this study.

## Supporting Information Available

Figures and raw thermochemistry and noncovalent interactions data supporting our analysis (PDF)
