# Supplementary material for: An Analysis of Regularized Second-Order Energy Expressions in the Context of Post-HF and KS-DFT Calculations: What Do We Gain and What Do We Lose?
Source: J Chem Theory Comput. 2025 Mar 6;21(6):2928–41. doi: 10.1021/acs.jctc.4c01547 (PMC11948328; doi:10.1021/acs.jctc.4c01547)
Supplement: Supplementary file 1 — ct4c01547_si_001.pdf [file ct4c01547_si_001.pdf]

Supporting information for "An analysis of regularized second-order energy expressions in the context of post-HF and KS-DFT calculations. What do we gain, and what do we lose?"

Igor Sawicki, Vincenzo Triglione, Subrata Jana, and Szymon Śmiga*

Institute of Physics, Faculty of Physics, Astronomy and Informatics, Nicolaus Copernicus University in Toruń, ul. Grudziądzka 5, 87-100 Toruń, Poland

E-mail: szsmiga@fizyka.umk.pl

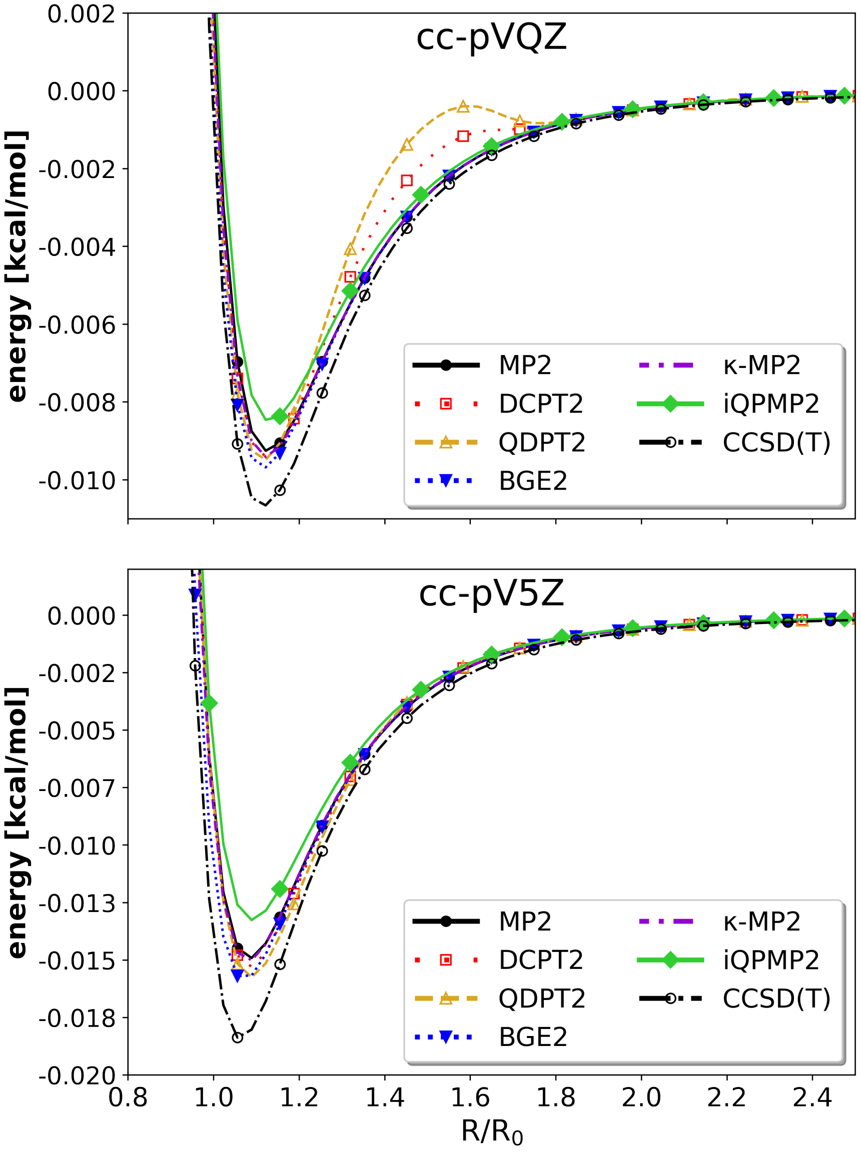

Figure S1: Interaction energy curve for HeNe dimer, obtained with few regularized second-order energy expressions in cc-pVQZ (top) and cc-pV5Z (bottom) basis sets on top of HF reference orbitals. All calculations were corrected with basis set superposition error correction.

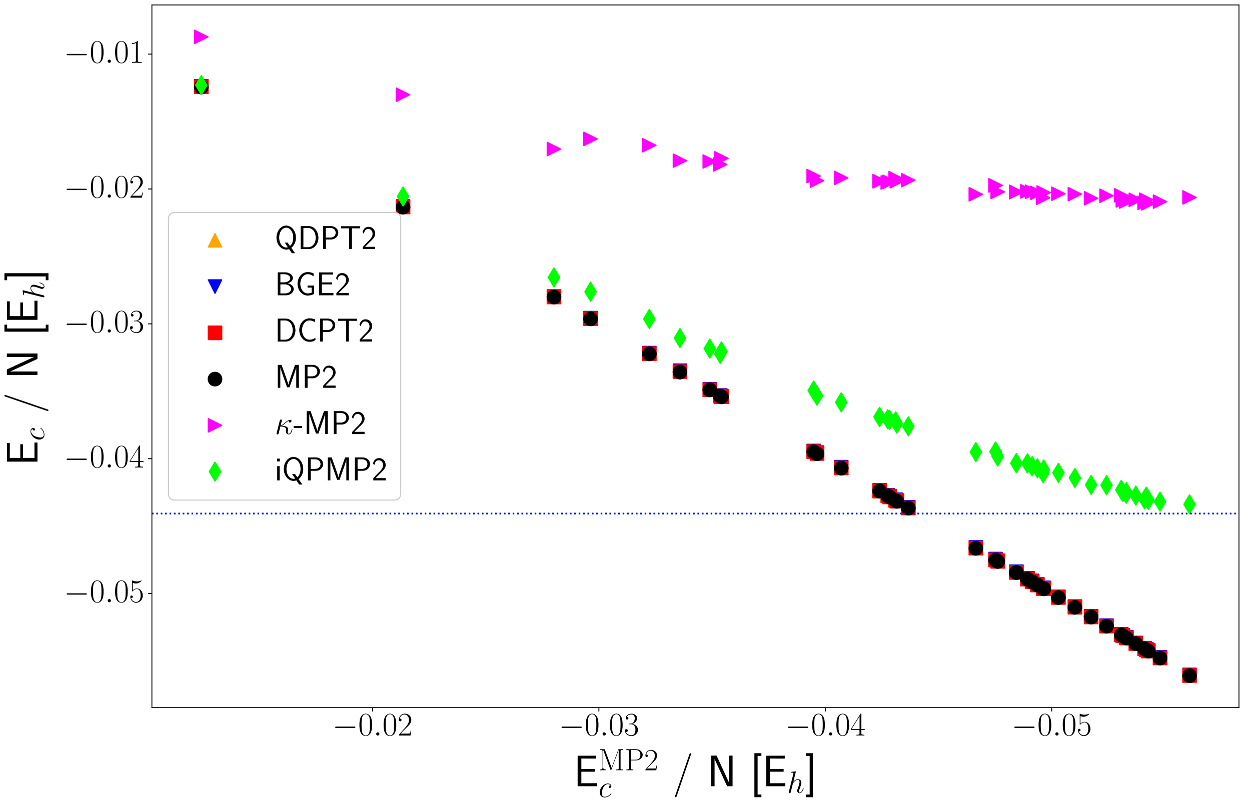

Figure S2: The correlation energy per electron for homogeneous electron gas model computed for few regularized MP2 energy expressions in the function of MP2 correlation energy per electron. Deviation from linearity in this plot indicates convergence or slower divergence than MP2. The calculations have been performed using the HF reference state. Each point corresponds to a single HEG calculation for $N \in (14, 3006)$ electrons in a finite cubic simulation cell with the Wigner-Seitz radius of $r_s = 2.07$ a.u. For the basis set details, see Ref. 37. The horizontal line indicates the exact HEG correlation energy value computed using PW92 correlation energy expression at CBS limit.

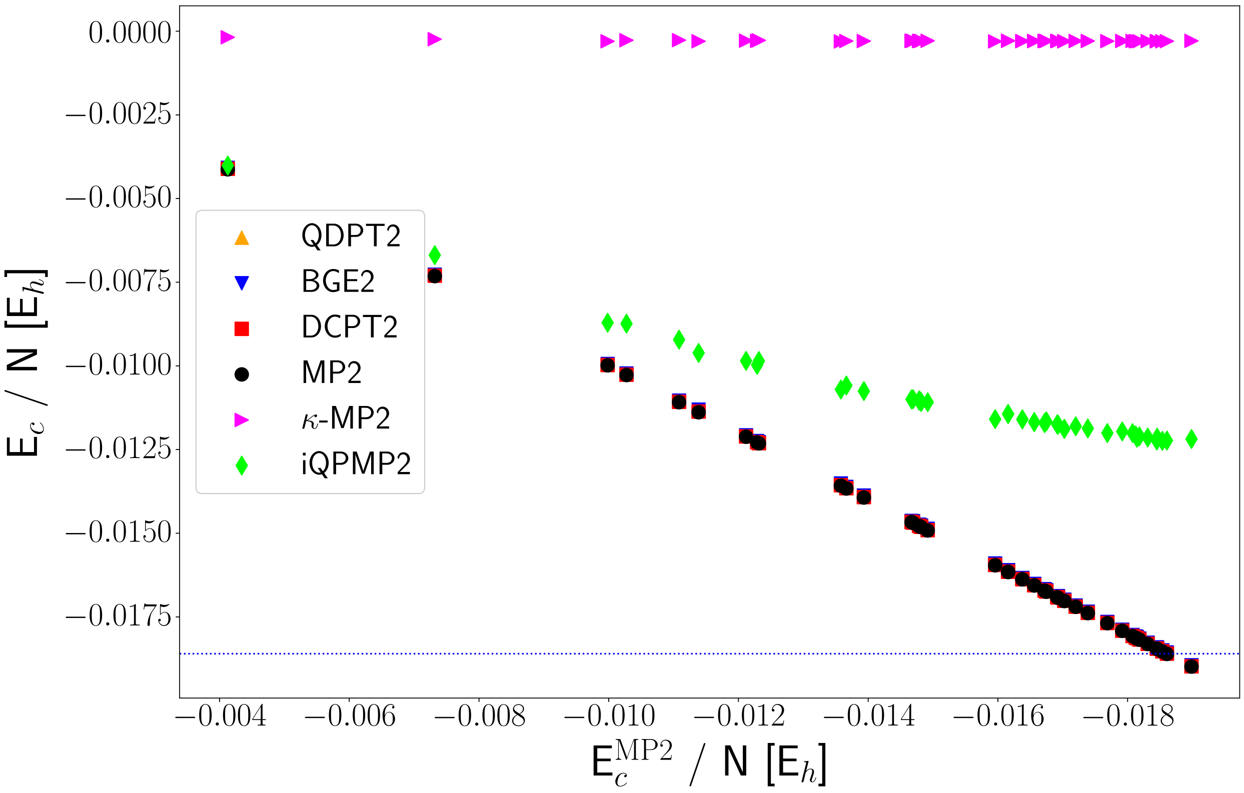

Figure S3: The correlation energy per electron for homogeneous electron gas model computed for few regularized MP2 energy expressions in the function of MP2 correlation energy per electron. Deviation from linearity in this plot indicates convergence or slower divergence than MP2. The calculations have been performed using the HF reference state. Each point corresponds to a single HEG calculation for $N \in (14, 3006)$ electrons in a finite cubic simulation cell with the Wigner-Seitz radius of $r_s = 10.0$ a.u. For the basis set details, see Ref. 37. The horizontal line indicates the exact HEG correlation energy value computed using PW92 correlation energy expression at CBS limit.

Table S1: Mean absolute errors (in kcal/mol) for the benchmark tests obtained using PBE-QIDH functional and various regularized second-order energy expressions. The last line reports the total mean absolute error (TMAE).

	MP2	DCPT2	QDPT2	BGE2	κ-MP2	iQPMP2
Main group thermochemistry (MGT)						
AE6	5.06	5.06	5.08	5.13	6.48	5.00
G2/148	5.41	5.42	5.45	5.52	6.77	5.61
G21EA	3.01	3.01	3.01	3.08	5.06	3.30
G21IP	2.64	2.65	2.69	2.71	3.44	2.81
PA26	2.04	2.04	2.04	2.03	3.02	2.17
BH76RC	1.52	1.54	1.54	1.56	1.58	1.50
SIE4x4	3.33	3.33	3.33	3.39	2.31	3.08
Barrier heights (BH)						
BH6	0.85	0.86	0.86	0.89	1.14	0.82
HTBH38	0.96	0.96	0.96	0.94	1.48	0.98
NHTBH38	1.78	1.78	1.78	1.79	2.55	1.81
Non-covalent interactions (NCI)						
HB6	0.29	0.30	0.30	0.44	0.36	0.30
DI6	0.25	0.25	0.24	0.25	0.26	0.24
CT7	0.40	0.41	0.43	0.47	0.31	0.35
PPS5	0.36	0.32	0.28	0.22	0.50	0.41
WI7	0.19	0.18	0.18	0.16	0.20	0.15
TMAE	1.87	1.87	1.88	1.91	2.36	1.90

Table S2: Mean absolute errors (in kcal/mol) for the benchmark tests obtained using B2-PLYP functional and various regularized second-order energy expressions. The last line reports the total mean absolute error (TMAE).

	MP2	DCPT2	QDPT	BGE2	κ-MP2	iQPMP2
Main group thermochemistry (MGT)						
AE6	1.18	1.18	1.18	1.16	4.89	1.49
G2/148	2.69	2.69	2.67	2.68	6.44	3.31
G21EA	2.57	2.57	2.57	2.60	4.68	3.26
G21IP	2.38	2.39	2.41	2.45	3.42	2.46
PA26	1.16	1.16	1.16	1.17	2.33	1.29
BH76RC	0.86	0.87	0.87	0.90	0.99	0.90
SIE4x4	9.84	9.84	9.83	9.89	8.56	9.60
Barrier heights (BH)						
BH6	2.19	2.19	2.20	2.28	1.14	2.03
HTBH38	1.78	1.78	1.79	1.87	1.23	1.63
NHTBH38	2.51	2.52	2.53	2.63	1.53	2.30
Non-covalent interactions (NCI)						
HB6	0.30	0.30	0.29	0.25	0.28	0.32
DI6	0.40	0.39	0.37	0.35	0.35	0.42
CT7	0.39	0.41	0.43	0.50	0.25	0.31
PPS5	1.38	1.32	1.25	0.93	1.43	1.42
WI7	0.23	0.23	0.23	0.20	0.24	0.24
TMAE	1.99	1.99	1.99	1.99	2.52	2.07

Table S3: Mean absolute errors (in kcal/mol) for the benchmark tests obtained using RSX-QIDH functional and various regularized second-order energy expressions. The last line reports the total mean absolute error (TMAE).

	MP2	DCPT2	QDPT	BGE2	κ-MP2	iQPMP2
Main group thermochemistry (MGT)						
AE6	5.93	5.94	5.93	6.07	8.43	6.64
G2-148	6.88	6.89	6.90	6.99	9.20	7.64
G21EA(QZVPD)	3.86	3.86	3.87	3.95	5.35	4.09
G21IP	3.18	3.19	3.21	3.23	3.76	3.31
PA26	2.51	2.51	2.51	2.52	3.33	2.64
BH76RC	1.53	1.54	1.54	1.56	1.65	1.54
SIE4x4	2.30	2.30	2.30	2.20	3.07	2.49
Barrier heights (BH)						
BH6	1.59	1.59	1.59	1.54	2.02	1.67
HTBH38	1.91	1.91	1.91	1.83	2.66	2.05
NHTBH38	3.10	3.09	3.08	2.98	3.97	3.21
Non-covalent interactions (NCI)						
HB6	0.27	0.28	0.28	0.33	0.32	0.27
DI6	0.31	0.30	0.29	0.29	0.37	0.33
CT7	0.44	0.42	0.40	0.36	0.69	0.50
PPS5	0.45	0.43	0.40	0.19	0.56	0.49
WI7	0.19	0.19	0.18	0.16	0.20	0.19
TMAE	2.30	2.30	2.29	2.28	3.04	2.47

Table S4: Mean absolute errors (in kcal/mol) for the benchmark tests obtained using BL1p functional and various regularized second-order energy expressions. The last line reports the total mean absolute error (TMAE).

	MP2	DCPT2	QDPT	BGE2	κ-MP2	iQPMP2
			Main group thermochemistry (MGT)			
AE6	1.61	1.61	1.60	1.62	5.74	4.59
G2/148	3.97	3.98	3.98	4.08	7.41	6.55
G21EA	3.96	3.96	3.96	4.00	6.63	4.55
G21IP	2.51	2.51	2.51	2.54	3.64	2.90
PA26	1.10	1.10	1.10	1.09	2.02	1.32
BH76RC	1.27	1.28	1.28	1.29	1.35	1.17
SIE4x4	1.53	1.53	1.53	1.70	1.69	1.34
			Barrier heights (BH)			
BH6	2.57	2.57	2.56	2.47	3.43	2.80
HTBH38	2.99	2.98	2.97	2.81	3.97	3.23
NHTBH38	4.54	4.53	4.52	4.36	5.29	4.59
			Non-covalent interactions (NCI)			
HB6	0.16	0.16	0.17	0.32	0.24	0.18
DI6	0.18	0.17	0.17	0.21	0.28	0.24
CT7	0.17	0.16	0.15	0.25	0.35	0.24
PPS5	0.49	0.49	0.51	0.98	0.48	0.37
WI7	0.13	0.13	0.12	0.13	0.14	0.14
TMAE	1.81	1.81	1.81	1.86	2.85	2.28

Table S5: Mean absolute errors (in kcal/mol) for the benchmark tests obtained using PBE-QIDH (κ_{opt}=3.18 E_h^{-1}, ξ_2^{iQPMP2}=0.418) functional for κ-MP2 and iQPMP2 with optimized parameters (κ and weight of second order correlation in XC, respectively). The last line reports the total mean absolute error (TMAE).

	κ-MP2-opt	iQPMP2-opt
Main group thermochemistry (MGT)		
AE6	4.94	4.27
G2/148	5.73	5.73
G21EA	3.70	2.47
G21IP	2.93	2.26
PA26	2.27	1.68
BH76RC	1.53	1.58
SIE4x4	3.00	4.08
Barrier heights (BH)		
BH6	0.83	1.19
HTBH38	0.99	0.96
NHTBH38	1.95	1.76
Non-covalent interactions (NCI)		
HB6	0.31	0.27
DI6	0.24	0.24
CT7	0.35	0.48
PPS5	0.40	0.28
WI7	0.14	0.12
TMAE	1.95	1.82

Table S6: Mean absolute errors (in kcal/mol) for a few benchmark sets obtained using PBE-QIDH functional and various regularized second-order energy expressions. All calculations have been performed in the def2-QZVP basis set without BSSE correction. The optimal parameters in κ-MP2 and iQPMP2 are κ_{opt}=3.18 E_h^{-1} and $\xi_{2_{opt}}$=0.418, respectively.

	MP2	κ-MP2	κ-MP2-opt	iQPMP2	iQPMP2-opt
S22	0.74	1.06	0.83	0.85	0.58
S66	0.81	1.05	0.87	0.88	0.67
A24	0.26	0.33	0.28	0.28	0.22
X31	0.61	0.76	0.65	0.65	0.52

Table S7: Mean absolute errors (in kcal/mol) for a few benchmark sets obtained using B2-PLYP functional and various regularized second-order energy expressions. All calculations have been performed in the def2-QZVP basis set without BSSE correction.

	MP2	DCPT2	QDPT2	BGE2	κ-MP2	iQPMP2
S22	1.79	1.76	1.74	1.49	2.02	1.87
S66	1.59	1.57	1.55	1.39	1.78	1.65
A24	0.50	0.48	0.46	0.39	0.56	0.27
X31	0.91	0.90	0.89	0.84	1.08	0.95

Table S8: Mean absolute errors (in kcal/mol) for a few benchmark sets obtained using RSX-QIDH functional and various regularized second-order energy expressions. All calculations have been performed in the def2-QZVP basis set without BSSE correction.

	MP2	DCPT2	QDPT2	BGE2	κ-MP2	iQPMP2
S22	0.92	0.92	0.90	0.81	1.15	1.01
S66	0.96	0.96	0.94	0.87	1.13	1.01
A24	0.34	0.26	0.33	0.28	0.39	0.28
X31	0.72	0.72	0.71	0.69	0.83	0.74

Table S9: Mean absolute errors (in kcal/mol) for a few benchmark sets obtained using BL1p functional and various regularized second-order energy expressions. All calculations have been performed in the def2-QZVP basis set without BSSE correction.

	MP2	DCPT2	QDPT2	BGE2	κ-MP2	iQPMP2
S22	0.24	0.25	0.25	0.34	0.48	0.42
S66	0.34	0.33	0.33	0.33	0.59	0.51
A24	0.24	0.18	0.17	0.21	0.27	0.24
X31	0.35	0.30	0.30	0.30	0.44	0.35
